# Effects of Cadmium and Mercury on the Upper Part of Skeletal Muscle Glycolysis in Mice

**DOI:** 10.1371/journal.pone.0080018

**Published:** 2014-01-28

**Authors:** Maria José Ramírez-Bajo, Pedro de Atauri, Fernando Ortega, Hans V. Westerhoff, Josep Lluis Gelpí, Josep J. Centelles, Marta Cascante

**Affiliations:** 1 Department of Biochemistry and Molecular Biology and IBUB, Faculty of Biology, Universitat de Barcelona, Barcelona, Spain; 2 Institut d'Investigacions Biomèdiques August Pi i Sunyer (IDIBAPS), Barcelona, Spain; 3 Department of Molecular Cell Physiology, BioCentrum Amsterdam, Faculty of Biology, Vrije Universiteit, Amsterdam, The Netherlands; 4 Manchester Interdisciplinary Biocentre-3.018, School of Chemical Engineering and Analytical Science, The University of Manchester, Manchester, United Kingdom; 5 Synthetic Systems Biology, SILS and NISB, University of Amsterdam, Amsterdam, The Netherlands; University Paris Diderot-Paris 7, France

## Abstract

The effects of pre-incubation with mercury (Hg^2+^) and cadmium (Cd^2+^) on the activities of individual glycolytic enzymes, on the flux and on internal metabolite concentrations of the upper part of glycolysis were investigated in mouse muscle extracts. In the range of metal concentrations analysed we found that only hexokinase and phosphofructokinase, the enzymes that shared the control of the flux, were inhibited by Hg^2+^ and Cd^2+^. The concentrations of the internal metabolites glucose-6-phosphate and fructose-6-phosphate did not change significantly when Hg^2+^ and Cd^2+^ were added. A mathematical model was constructed to explore the mechanisms of inhibition of Hg^2+^ and Cd^2+^ on hexokinase and phosphofructokinase. Equations derived from detailed mechanistic models for each inhibition were fitted to the experimental data. In a concentration-dependent manner these equations describe the observed inhibition of enzyme activity. Under the conditions analysed, the integral model showed that the simultaneous inhibition of hexokinase and phosphofructokinase explains the observation that the concentrations of glucose-6-phosphate and fructose-6-phosphate did not change as the heavy metals decreased the glycolytic flux.

## Introduction

The concentration of heavy metals in the environment has increased significantly over recent decades [1]. Much of this is due to increased human activity such as industrial activity, traffic, smelting, fossil fuel combustion and agriculture [2]. These metals cannot be degraded and their accumulation in the food chain produces human health risks and ecological disturbances [3], [4]. The toxicity of each metal depends on many factors, including the duration, quantity and exposure method, as well as the chemical form in which it exists. Once assimilated by the body, metals can cause a variety of cytotoxic reactions [5], [6], [7], [8], [9]. They may affect essential metabolic pathways in cells [7] or lead to the production of reactive oxygen species (ROS), which affect various cellular processes, including the functioning of the membrane system [10]. Many studies have reported the biological implications of metal toxicity in metabolic and associated physiological processes [11], [12], [13], [14], including the effect of cadmium, mercury and copper on the upper part of glycolysis or on the process of tubulin polymerization [15], [16], [17]. The mechanisms of toxicity of these heavy metals include the interaction with proteins due to the high affinity of the former for the free electron pairs in cysteine SH groups [7], [10], [18], [19]. These groups can determine the structure and conformation of the enzyme or engage in catalysis at the active centre of the enzyme.

Metabolic control analysis (MCA) has been used extensively to quantify enzyme control on system variables, usually steady-state fluxes and metabolite concentrations [20], [21], [22], [23], [24]. This control is evaluated by means of control coefficients, which are sensitivity coefficients of these system variables in terms of activity changes of one enzyme, e.g. *C^J^_E_ = d ln (flux)/d ln (enzyme activity)*. In previous works, we observed that, in extracts of mouse skeletal muscle, hexokinase (HK) and phosphofructokinase (PFK) shared control of the metabolic flux in the upper part of glycolysis [15], [25]. Also, we characterized the irreversible inhibitory effects of pre-incubation with copper and we identified HK and PFK as the main targets of copper [15]. This led us to propose that other heavy metals affect metabolic functions through their irreversible effects on HK and PFK. The main aim of this study is to test this hypothesis by identifying the flux control mechanism and sites of action of two other heavy metals, Hg^2+^ and Cd^2+^, in the upper part of glycolysis. We therefore pre-incubated mouse muscle extracts with these toxic agents and then characterized the effect of increasing the concentrations of both on activities of individual enzymes, flux of the overall multi-enzymatic pathway, intermediate metabolite concentrations and flux control coefficients.

## Materials and Methods

### Chemicals

ATP (sodium salt), NADP^+^, NADH, glucose (Glc), glucose-6-phosphate (G6P), fructose-6-phosphate (F6P), fructose-1,6-bisphosphate (FBP), HK, aldolase (ALD), triose-phosphate isomerase (TPI), glucose-6-phosphate dehydrogenase (G6PDH), α-glycerol-3-phosphate dehydrogenase (GDH), PFK, phosphocreatine (PC), creatine kinase (CK), Cd(NO_3_)_2_, Hg(NO_3_)_2_. HEPES and 3-(N-morpholino)propanesulphonic acid (MOPS) were purchased from Sigma-Aldrich (St. Louis, MO). The protease inhibitor (Pefabloc®) was purchased from Boehringer Ingelheim GmbH (Germany). Bio-Rad Protein Assay was purchased from Bio-Rad Laboratories GmbH (Germany). All other chemicals (analytical grade) were purchased from Panreac (Spain).

### Preparation of muscle extracts

After cervical dislocation and confirmed the mice death, leg muscle of 8- to 16-week-old C57BL/6 mice (IFFA Credo, Spain) was minced with scissors and 1.5 g muscle was homogenized in 3 ml of ‘standard buffer’ (50 mM HEPES, pH 7.4 containing 100 mM KCl, 10 mM NaH_2_PO_4_ and 10 mM MgCl_2_). 50 µl of Pefabloc®/3 ml homogenate was added. Homogenization was carried out in liquid nitrogen with a mortar. The homogenate was centrifuged at 31000×g for 30 min and the supernatant was filtered through Waterman paper. All procedures were carried out at 4°C. The protein concentration of the extract was determined using the Bradford method (Bio-Rad Laboratories GmbH, Germany) [26]. The extract was diluted to adjust the protein concentration to 1.2 mg/ml in all of the experiments. The protocol was supervised and approved by the Committee on the Ethics of Animal Experiments of the University of Barcelona (CEEA: Comité Ètic d'Experimentació Animal).

### Determination of the flux from glucose to triose phosphates

The steady-state fluxes were measured in standard buffer at 37°C by coupling the reaction with an excess of the auxiliary enzymes TPI and GDH. The final concentrations in the cuvette were 2 mM NADH, 2 mM MgATP, 10 mM Glc, 20 mM PC, 3 U/ml CK, 3.5 U/ml TPI and 0.5 U/ml GDH. NADH consumption was monitored at 385 nm (ε^385 nm^_NADH_ = 0.75 mM^−1^ cm^−1^), as described by Puigjaner *et al.* [25], using a Shimadzu UV-2101PC Spectrophotometer with 1-cm light path cells. These assay conditions are not quite representative for mouse muscle *in vivo*, but in view of the considerable amount of consensus building required to achieve proper *in vivo* standard conditions [27], we here reverted to conditions that are not far off from the *in vivo* state and that our previous work [15], [25] has shown to work well.

Extracts were pre-incubated with Cd(NO_3_)_2_ (0–7 µM) and Hg(NO_3_)_2_ (0–10 µM) in standard buffer at 37°C for 60 min. The reaction was then started by adding 100 µl of the reaction mixture to 900 µl of the pre-incubated extract.

### Determination of the metabolite concentrations

The metabolite concentrations were determined when, in the above assay, the NADH consumption proceeded at a constant rate. The reaction was stopped at different intervals by adding ice-cold HClO_4_ to a final concentration of 10% and neutralized to pH 7.0 with KOH/MOPS (6M/0.6M). After 10 min, the precipitate was removed by centrifugation for 10 min at 14000×g. The supernatants were used for the enzymatic determination of G6P and F6P, in accordance with Bergmeyer [28].

### Modulation of steady-state flux and metabolite concentration by external enzymes and determination of flux control coefficients

The steady-state flux was measured when commercial enzyme (HK) or partially purified enzyme (PFK) was added to the extract in order to determine the control coefficients using classical titration analysis. In each case the appropriate amounts of enzyme were added to the extract pre-incubated with Cd(NO_3_)_2_ or Hg(NO_3_)_2_ in a standard buffer for 60 min at 37°C. The reaction was started with the addition of 100 µl of the reaction mixture containing different amounts of commercial enzyme or partially purified enzyme to 900 µl of the pre-incubated extract. Since a large quantity of exogenous enzyme was added, it was not necessary to determine the enzymatic activity with accuracy. Flux control coefficients were estimated using the Small and Kacser method for large changes in enzyme activity [29], for the conversion of Glc to triose-phosphates (TrP).

### Measurements of activities of individual enzymes

The maximal catalytic activities of HK, glucose-6-phosphate isomerase (GPI), PFK and ALD were measured under substrate saturation in the standard buffer with 900 µl of pre-incubated extract mixture (the pre-incubated extract mixture contained Cd(NO_3_)_2_ (0–7 µM) or Hg(NO_3_)_2_ (0–10 µM) and, for flux control coefficient determinations, the appropriate amounts of commercial or partially purified enzymes) at 37°C. GPI and ALD individual activities were measured in accordance with Bergmeyer's methods [28]. PFK activity was measured in accordance with the method of Brand and Söling [30]. HK activity was measured as described by Grossbard and Schimke [31].

### Computer modelling of the pathway

**Figure 1:**
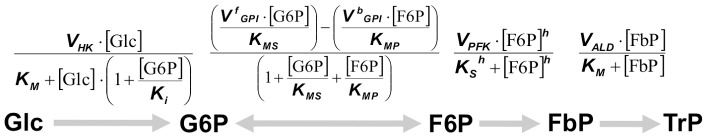
Scheme of the kinetic model. The scheme, equations and parameter values correspond to the kinetic model published previously [25]. Parameters for HK: *K_M_* = 0.40 mM, *K_i_* = 0.11 mM. Parameters for GPI: *V^f^_GPI_* = 12474 nmol mg prot^−1^ min^−1^, *V^b^_GPI_* = 18125 nmol mg prot^−1^ min^−1^, *K_MS_* = 0.48 mM, *K_MP_* = 0.27 mM. Parameters for PFK: *K_S_* = 0.061 mM, *h* = 1.47. Parameters for ALD: *V_ALD_* = 6000 nmol mg prot^−1^ min^−1^, *K_M_* = 0.13 mM. Limiting rates for HK (*V_HK_*) and PFK (*V_PFK_*) decrease at increasing values for Hg^2+^ and Cd^2+^ following equations (1) and (2), respectively for Hg^2+^ and Cd^2+^, with *V^0^_HK_* = 63.0 nmol mg prot^−1^ min^−1^ and *V^0^_PFK_* = 434 nmol mg prot^−1^ min^−1^.

Two detailed rate- plus balance-equation models were developed based on our previously published paper [25]. Network and equations are described in Fig. 1. A first model was adapted to change the limiting rates (maximal velocities) in the presence of various concentrations of Hg^2+^. A second model was adapted to change the limiting rates in the presence of various concentrations of Cd^2+^.

### Molecular modelling analysis

Models for the 3D structures of mouse HK, isoenzyme II (UniprotKb entry O08528) and PFK (UniprotKb entry P47857) where obtained from the SwissModel repository [32] using the template structures 2NZT (human HK II, 92% sequence identity) and 3O8L (rabbit muscle PFK, 96% sequence identity), respectively. Ligands bound to these and other homologous structures were used to assess the position of Cys residues in relation to active or regulatory sites. The solvent accessible surface was determined using the program NACCESS [33] and structures were analysed visually using Pymol v. 1.3.

## Results

### Inhibition of glycolytic enzymes by Hg^2+^ and Cd^2+^

In order to establish the experimental conditions, we first examined the time dependence of the inhibition of HK, GPI, PFK and ALD by Hg^2+^ and Cd^2+^. Mouse muscle extracts were pre-incubated with different Hg^2+^ concentrations (0–10 µM) and Cd^2+^ concentrations (0–7 µM) for up to 1 h at 37°C. After the incubation time, the activities of individual enzymes were measured. In the range of Hg^2+^ and Cd^2+^ concentrations tested, HK activity and PFK activity decreased with pre-incubation time until they reached a limit value after 1 h. The GPI activity and ALD activity remained constant during this time interval for all Hg^2+^ and Cd^2+^ concentrations tested. In the following experiments, pre-incubation times of 1 h were used.

**Figure 2:**
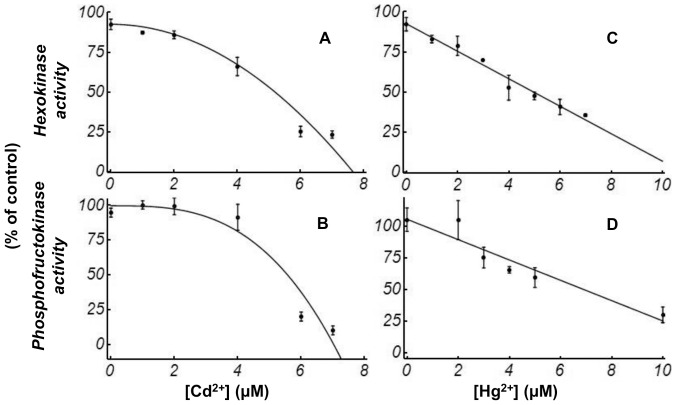
HK and PFK activities. Effect of increasing pre-incubating concentrations of Cd^2+^ (A,B) or Hg^2+^ (C,D) on individual HK activity (A,C) and PFK activity (B,D). The length of the error bar on either side of the mean values represents one standard deviation (SD). Thus, all values are mean ±1 SD. Solid lines represent the fitting of the activities of HK (A and C) or PFK (B and D) to the respective equations (A and B for equation (2), C and D for equation (1)).

In the control samples without heavy metals, the decrease in activities of individual enzymes was around 5%. HK activity was inhibited by Cd^2+^ and Hg^2+^ in a concentration-dependent manner (Fig. 2A and Fig. 2C, respectively). The IC_50_ were estimated as the concentration of Cd^2+^ or Hg^2+^ for half activity. The IC_50_ for HK were around 5 µM for Cd^2+^ or Hg^2+^. Cd^2+^ and Hg^2+^ also inhibited PFK activity, virtually to the same extent and at the same concentrations as they inhibited HK (Fig. 2B and Fig. 2D). In this case, the IC_50_ was around 5 µM for Cd^2+^ and 6 µM for Hg^2+^.

### Effects of Hg^2+^ and Cd^2+^ on the steady-state flux of triose phosphate production and on steady-state intermediate metabolite concentrations

**Figure 3:**
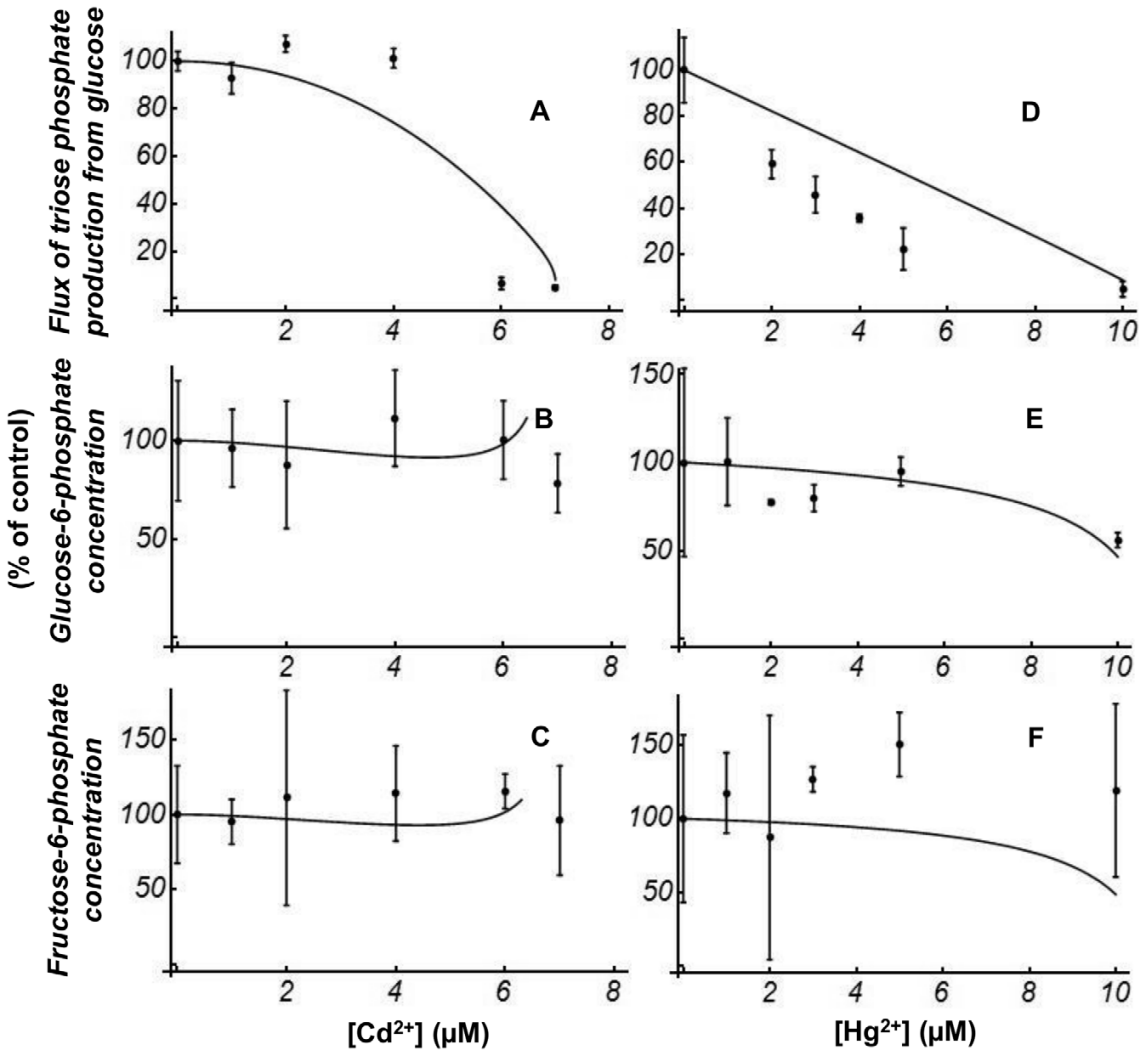
Steady state flux and G6P and F6P concentrations. Decrease of flux of steady-state TrP production from Glc (A,D) and maintenance of G6P (B,E) and F6P (C,F) steady-state concentrations, in the presence of different pre-incubating concentrations of metals. All values are mean ±1 SD. Solid lines represent the steady-state concentration and flux values calculated by simulation using the complete model as described in Fig. 1 and including equation (1) and equation (2). Experimental and calculated values are independently scaled to be 100% at zero concentration of Cd^2+^ and Hg^2+^.

The results of the effects of Cd^2+^ and Hg^2+^ on the steady-state flux of TrP production and the steady state of G6P and F6P concentrations are shown in Fig. 3. The IC_50_ of the steady-state flux of TrP production from Glc was around 5 µM for Cd^2+^ and around 3.5 µM for Hg^2+^. These values are close to the corresponding IC_50_ values for HK and PFK, which would suggest that these enzymes had a high level of control over the measured flux. The distribution of the control of the flux of TrP production can be estimated by flux control coefficients [23]. These control coefficients were determined experimentally (for additional details see Materials & Methods). In the absence or presence of the heavy metals ([Cd^2+^] = 5 µM, or [Hg^2+^] = 3.5 µM), the resulting flux control coefficients for HK (*C^J^_HK_*) and PFK (*C^J^_PFK_*) were in the range 0.91–0.96 and 0.11–0.25, respectively. The distribution of control among the enzymes was not significantly changed by the toxic agents, nor were the steady-state G6P and F6P concentrations when the extract was pre-incubated with different concentrations of Cd^2+^ or Hg^2+^ (Fig. 3).

### Mathematical model of irreversible inhibition

The measured decreases in activities of individual enzymes resulting from inhibition by Hg^2+^ and Cd^2+^ were embedded in a mathematical model. A model describing the concentrations and flux through the upper part of glycolysis as a function of rate laws of the individual enzymes in mouse muscle extracts had previously been developed [25]. As described in Fig. 1, HK follows a simple Michaelis-Menten kinetics equation and PFK a Hill equation in this model. The limiting rates (*V*) for HK, GPI, PFK and ALD are such that the flux control coefficients for HK and PFK were 0.85 and 0.15, respectively, which are within the observed ranges.

**Figure 4:**
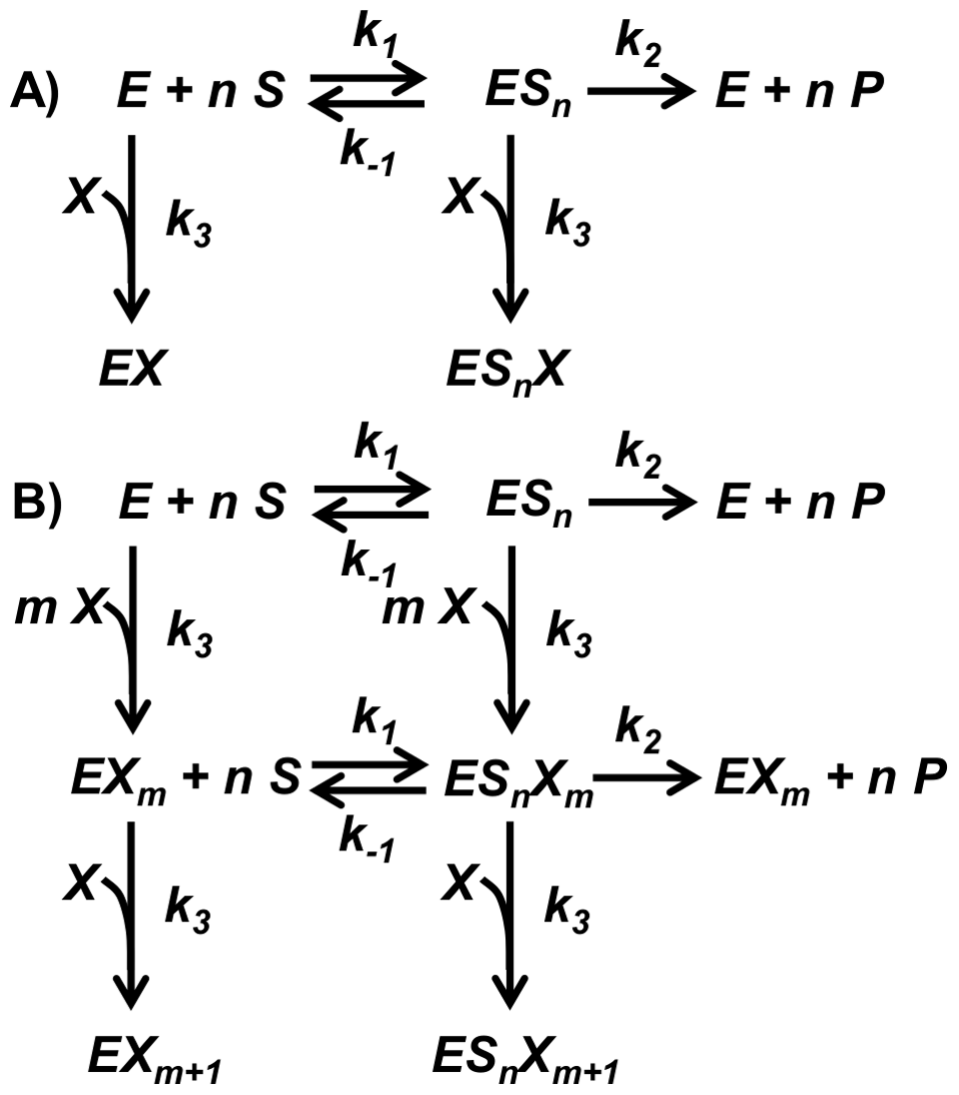
Schemes for mechanisms of enzyme irreversible inhibition. Mechanisms of irreversible inhibition of HK activity and PFK activity by Hg^2+^ (A) and Cd^2+^ (B). *E* represents HK or PFK, *S* the respective substrate, *ES_n_* the enzyme-substrate complex (HK follows a Michaelis-Menten equation (n = 1), whilst PFK is an allosteric enzyme (n>1)). *P* represents the products of the respective reactions, *X* the metal ions (Cd^2+^ or Hg^2+^), whilst *n* is the number of substrate binding sites and *m*+1 is the number of metal ion (*X*) molecules that can be bound irreversibly to the enzyme. The best agreement to the experimental results was obtained with m = 1 for HK and m = 2 for PFK.

With respect to the irreversible inhibition by Hg^2+^ or Cd^2+^ on HK activity and PFK activity, we proposed the mechanistic models depicted in Fig. 4. For Hg^2+^, a first mechanistic model takes into account that Hg^2+^ only affects *V* and not the *K_M_* or *K_S_*. The equations derived from scheme in Fig. 4A for HK and PFK inhibition by Hg^2+^ are presented in detail in Appendix S1. The resulting *V* dependency on metal concentrations is detailed in the following equation:
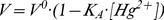
(1)where *V^0^* refers to the limiting rate in the absence of Hg^2+^ and *K_A_* is the apparent inactivation constant. The model predicts that the inactivation is linearly related to the added concentration of Hg^2+^. This is confirmed by Fig. 2C and 2D: a straight line fits the experimental results, which led us to estimate that the *K_A_* was 92 mM^−1^ for HK and 76 mM^−1^ for PFK.

The obtained experimental data on the irreversible inhibition exerted by Cd^2+^ on HK activity and PFK activity (Fig. 2A and 2B) cannot be fitted using the model in Fig. 4A, which considers a 1∶1 stoichiometry for the binding between the metal and the enzyme. For this case, we considered an alternative mechanistic model (see Fig. 4B and Appendix S1). Cd^2+^ binds to *m+1* sites, and only when this bind is complete, inactivation takes place. This leads to the following expression for the *V* dependence on the concentration of the added inhibitor:
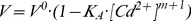
(2)where *m* is 1 for HK and 2 for PFK, *V^0^* refers to the limiting rate in the absence of Cd^2+^ and *K_A_* is the apparent inactivation constants of Cd^2+^. Fig. 2A and 2B show the mathematical fitting for Cd^2+^ for both enzymes, leading us to estimate that the *K_A_* was 0.017 µM^−2^ for HK and 0.0029 µM^−3^ for PFK.

The substitution, in the mathematical model described in Fig. 1, of the *V* of HK (*V_HK_*) and PFK (*V_PFK_*) by the new Cd^2+^ or Hg^2+^ dependent expressions (equation (1) and equation (2)) reproduced the observed behaviour approximately, as shown in Fig. 3. This figure provides a direct comparison of the experimental data for maintenance of the metabolite concentrations and decrease of the flux, respectively, with the model predictions.

### Structural analysis of metal inhibition

**Figure 5:**
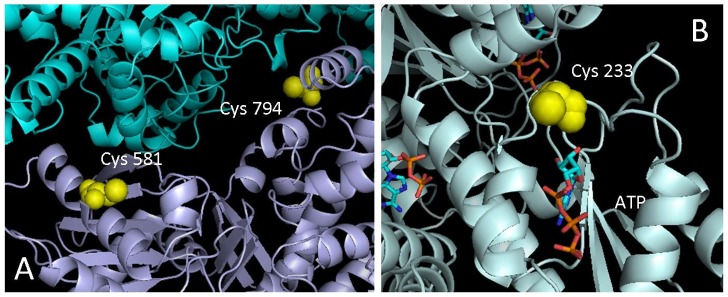
Position for most relevant candidates to irreversible inhibition. (A) Cys 581 and Cys 794 on intersubunit interface of HK, (B) Cys 233 near to ATP binding site on PFK.

Irreversible inhibition of enzymes by metals is usually attributed to the binding to amino acid residues (Cys and His being the most common) that are essential for enzyme activity or stability. To analyse the feasibility of metal inhibition from a structural point of view, 3D model structures of HK and PFK were examined to determine possible target residues for metal binding. Comparative models were obtained from ModBase (see Materials and Methods section) and inspected. Table 1 shows a summary of the results obtained. Candidate Cys residues were selected taking into account the exposure of the side chain, and their distance to relevant structures such as active sites and intersubunit contacts. In both cases, the analysis showed a significant number of candidate residues. Particularly relevant examples are Cys 581 and Cys 794 on HK in the intersubunit interface (Fig. 5A) and Cys 233 on PFK located at the ATP-binding site (Fig. 5B). Binding of such residues to metal ions greatly influences either the stability of the protein, in the case of HK, or the ability to bind ATP, for PFK, leading to irreversible inactivation. There are no Cys residues near the active sites of GPI and ALD.

**Table 1:** Summary of candidate Cys residues.

Protein	Residue	% exposed side chain	Location
HK	Cys 158	7.2	Glucose site
	Cys 517	46.5	Solvent exposed
	**Cys 581**	**6.1**	**Dimer interface**
	Cys 606	5.1	Glucose site
	Cys 717	26.7	Solvent exposed
	**Cys 794**	**89.0**	**Dimer interface**
PFK	**Cys 89**	**11.3**	**ATP site**
	**Cys 233**	**36**	**ATP site**

Summary of Cys residues with more than 30% exposed side chain or related to functionally relevant sites. Most relevant candidates for irreversible inhibition are shown in bold.

## Discussion

We previously analysed [25], [34] the first four steps of glycolysis in muscle extract, optimized the experimental system and characterized the control role and properties of HK and PFK in the upper part of glycolysis. In a subsequent study, we identified HK and PFK as the sites of action of Cu^2+^ [15]. Here we found the effects of Cd^2+^ and Hg^2+^ on the enzymes, flux and concentrations of the upper part of glycolysis in mouse muscle extract to be similar to our previous findings for copper. The similarity is especially strong for mercury and copper. Interestingly, the same equations [15] explain the irreversible inhibition observed experimentally, but with different parameters.

The analysis of Fig. 2 and 3 reveals that, for HK activity, PFK activity and the flux of TrP production, the relative decrease for increasing concentrations of Cd^2+^ or Hg^2+^ is similar. This implies similar IC_50_ values for the enzymes and the flux for Cd^2+^ or Hg^2+^. However, the patterns of decay for increasing concentrations of Cd^2+^ and Hg^2+^ are different, with a more linear decay for Hg^2+^. Also, the analysis of the G6P and F6P concentrations shows that they are unchanged in both incubations with Cd^2+^ and Hg^2+^ (Fig. 3), as was also previously reported for Cu^2+^ [15]. Both metabolites are the intermediaries among the reactions catalysed by HK and PFK, which are in rapid equilibrium through the reaction catalysed by GPI.

The analysis of the control coefficients *C^J^_HK_* and *C^J^_PFK_* calculated from the experimental data were in perfect agreement with the values previously reported in our studies with mouse muscle extracts [15], [25]. These were in the range 0.8–0.9 and 0.1–0.2, respectively. These data are also consistent with data reported in the literature for other cell types [35], [36]. According to the flux summation theorem of MCA, the sum of all flux control coefficients is one, and all control coefficients in a linear pathway are positive:
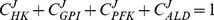
(3)This suggests that HK has the majority of control of the upper part of glycolysis in muscle extract and PFK has the remainder. There is no significant flux control in GPI and ALD. HK and PFK, the enzymes that share the control of the flux, are also the enzymes that are inhibited when pre-incubated with copper [15], cadmium and mercury, while GPI and ALD, which have no control, are almost unaffected by these heavy metals. The distribution of control is not changed significantly by the toxic agents, which is consistent with the observed maintenance of metabolite concentrations, since the elasticity coefficients [23], which cause the control coefficients to assume their values, are not affected by the inhibitors.

**Figure 6:**
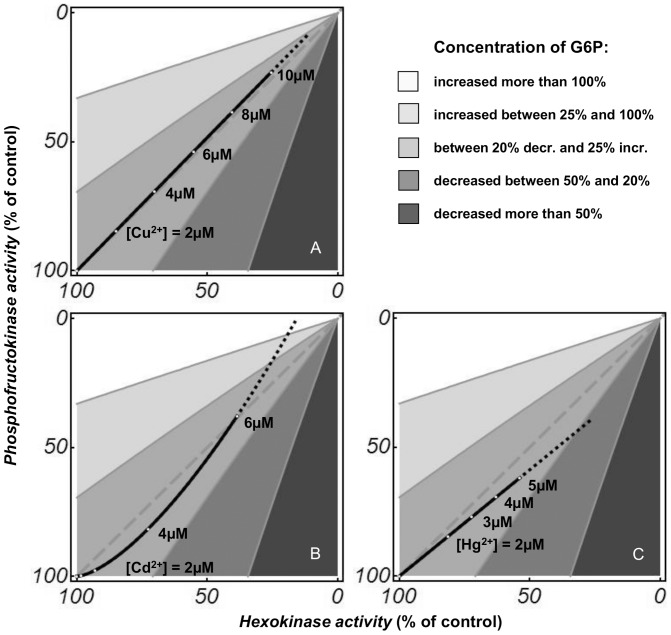
Model predictions showing the dependence of G6P concentrations on HK and PFK limiting rates. The proportional dysregulation predicts the broken grey line. The shades in the panels report the predicted G6P concentrations when the limiting rates of HK and PFK do not change proportionally. Solid black lines correspond to the model predictions for different levels of pre-incubation with Cu^2+^ (A), Cd^2+^ (B) and Hg^2+^ (C): they indicate how the metals change the limiting rates of the two enzymes and the consequent changes in metabolite concentrations. The same plot is for F6P, as G6P and F6P are in rapid equilibrium through the reaction catalysed by GPI.

The simultaneous decrease of the limiting rates of HK and PFK, the enzymes controlling the flux, is equivalent to a minimal implementation with only two enzymes of multisite modulation, which is an efficient way by which organisms achieve very large flux changes, but with only small concentration changes in internal metabolites [37], [38], [39]. Fig. 6 plots the dependence of the metabolite concentrations on HK and PFK limiting rates as shades of grey. Only a close-to-proportional reduction in HK and PFK limiting rates allows the relative concentrations of the intermediate metabolites to be maintained at a constant level. Our data suggest that for Cu^2+^ [15] the inhibition mechanisms of HK and PFK follow this proportionality almost perfectly (solid line in Fig. 6A). For Cd^2+^ (solid line in Fig. 6B) and Hg^2+^ (solid line in Fig. 6C), this does not differ a great deal. The higher similarities for Cu^2+^ and Hg^2+^ are not surprising because they fit the same equations that describe the inhibition of HK and PFK. A simple explanation could be that all of these heavy metals inhibit all enzymes through the same mechanism, based on their high affinity for the free electron pairs in cysteine SH groups, which are important in enzyme function. The structural analysis of HK and PFK (Table 1 and Fig. 5) revealed a significant number of candidate cysteine residues. Interestingly, the formation of organized structures could protect against these inhibitory effects, as it has been suggested for the complex association of proteins/enzymes to microtubules in neuronal systems [16], [17]. Indeed a combined enhancement of microtubule assembly and the flux of Glc conversion to TrP was observed in bovine brain extract, which also resulted in a decreased sensitivity to copper toxicity [16]. A subsequent analysis in bovine brain extract showed that tubulin diminished the inhibitory effect of not only copper but also cadmium and mercury on the upper part of glycolysis [17]. However, the flux-stimulating effect of microtubules in brain extract was not seen in mouse muscle extract [16].

The fact that pre-incubation with these heavy metals decreases the flux without altering the metabolite concentrations, at least in the upper part of glycolysis analysed, suggests that, at low pollutant concentration, its effects could be silent at metabolite levels, even though the glycolytic flux is affected. Depending on the turnover rate of the enzymes, the irreversible inhibition of heavy metals over the two key enzymes of glycolysis, HK and PFK, could have long-term cumulative effects. We would expect the level of the glycolytic enzymes to be controlled by the performance of the pathway, which suggests that irreversible damage to the enzymes would be repaired by the synthesis of new enzyme molecules. It is important to understand whether the heavy metal ion is detoxified when the faulty enzyme molecules that contain the heavy metal ion are degraded. The effects on glycolytic flux of adding different heavy metals, the regulatory mechanisms of enzyme synthesis and the detoxification mechanisms should be taken into account when defining the permitted concentrations of these metals in water.

## Supporting Information

Appendix S1 (DOCX)

## References

[1] Mas A, Azcue JM (1993) Metales en sistemas biológicos. Barcelona: Promociones y Publicaciones Universitarias, S.A.

[2] Pinto E, Sigaud-Kutner TCS, Leitão MAS, Okamoto OK, Morse D, et al (2003) Heavy metal-induced oxidative stress in algae. J Phycol 39: 1008–1018.

[3] Nordberg GF, Fowler BA, Nordberg M, Friberg L (2007) Handbook on the toxicology of metals. Amsterdam: Elsevier.

[4] Diaz S, Martin-Gonzalez A, Carlos Gutierrez J (2006) Evaluation of heavy metal acute toxicity and bioaccumulation in soil ciliated protozoa. Environ Int 32: 711–717. 16650895 10.1016/j.envint.2006.03.004

[5] Förstner U, Wittmann GTW (1979) Metal pollution in the aquatic environment. Berlin, Heidelberg, New York: Springer Verlag.

[6] Passow H, Rothstein A, Clarkson TW (1961) The general pharmacology of the heavy metals. Pharmacol Rev 13: 185–224. 13733187

[7] Vallee BL, Ulmer DD (1972) Biochemical effects of mercury, cadmium, and lead. Annu Rev Biochem 41: 91–128. 4570963 10.1146/annurev.bi.41.070172.000515

[8] Hilmy AM, Shabana MB, Daabees AY (1985) Bioaccumulation of cadmium: toxicity in Mugil cephalus. Comp Biochem Physiol C 81: 139–144. 2861040 10.1016/0742-8413(85)90105-7

[9] Jin YH, Clark AB, Slebos RJ, Al-Refai H, Taylor JA, et al (2003) Cadmium is a mutagen that acts by inhibiting mismatch repair. Nat Genet 34: 326–329. 12796780 10.1038/ng1172 PMC2662193

[10] Letelier ME, Lepe AM, Faundez M, Salazar J, Marin R, et al (2005) Possible mechanisms underlying copper-induced damage in biological membranes leading to cellular toxicity. Chem Biol Interact 151: 71–82. 15698579 10.1016/j.cbi.2004.12.004

[11] Kesseler A, Brand MD (1994) Localisation of the sites of action of cadmium on oxidative phosphorylation in potato tuber mitochondria using top-down elasticity analysis. Eur J Biochem 225: 897–906. 7957227 10.1111/j.1432-1033.1994.0897b.x

[12] Ciapaite J, Nauciene Z, Baniene R, Wagner MJ, Krab K, et al (2009) Modular kinetic analysis reveals differences in Cd2+ and Cu2+ ion-induced impairment of oxidative phosphorylation in liver. Febs J 276: 3656–3668. 19496816 10.1111/j.1742-4658.2009.07084.x

[13] Chassagnole C, Quentin E, Fell DA, de Atauri P, Mazat JP (2003) Dynamic simulation of pollutant effects on the threonine pathway in Escherichia coli. C R Biol 326: 501–508. 12886877 10.1016/s1631-0691(03)00098-2

[14] Strydom C, Robinson C, Pretorius E, Whitcutt JM, Marx J, et al (2006) The effect of selected metals on the central metabolic pathways in biology: A review. Water SA 32: 543–554.

[15] Jannaschk D, Burgos M, Centelles JJ, Ovadi J, Cascante M (1999) Application of metabolic control analysis to the study of toxic effects of copper in muscle glycolysis. FEBS Lett 445: 144–148. 10069389 10.1016/s0014-5793(99)00117-9

[16] Liliom K, Wagner G, Kovacs J, Comin B, Cascante M, et al (1999) Combined enhancement of microtubule assembly and glucose metabolism in neuronal systems in vitro: decreased sensitivity to copper toxicity. Biochem Biophys Res Commun 264: 605–610. 10529410 10.1006/bbrc.1999.1547

[17] Liliom K, Wagner G, Pacz A, Cascante M, Kovacs J, et al (2000) Organization-dependent effects of toxic bivalent ions microtubule assembly and glycolysis. Eur J Biochem 267: 4731–4739. 10903506 10.1046/j.1432-1327.2000.01526.x

[18] Jalilehvand F, Leung BO, Mah V (2009) Cadmium(II) complex formation with cysteine and penicillamine. Inorg Chem 48: 5758–5771. 19469490 10.1021/ic802278r PMC2878379

[19] Quig D (1998) Cysteine metabolism and metal toxicity. Altern Med Rev 3: 262–270. 9727078

[20] Kacser H, Burns JA (1973) The control of flux. Symp Soc Exp Biol 27: 65–104. 4148886

[21] Kacser H, Burns JA, Fell DA (1995) The control of flux. Biochem Soc Trans 23: 341–366. 7672373 10.1042/bst0230341

[22] Heinrich R, Rapoport TA (1974) A linear steady-state treatment of enzymatic chains. General properties, control and effector strength. Eur J Biochem 42: 89–95. 4830198 10.1111/j.1432-1033.1974.tb03318.x

[23] Fell DA (1997) Understanding the Control of Metabolism. London: Portland Press.

[24] Groen AK, Wanders RJ, Westerhoff HV, van der Meer R, Tager JM (1982) Quantification of the contribution of various steps to the control of mitochondrial respiration. J Biol Chem 257: 2754–2757. 7061448

[25] Puigjaner J, Raïs B, Burgos M, Comín B, Ovadi J, et al (1997) Comparison of control analysis data using different approaches: modelling and experiments with muscle extract. FEBS Lett 418: 47–52. 9414093 10.1016/s0014-5793(97)01347-1

[26] Bradford MM (1976) A rapid and sensitive method for the quantitation of microgram quantities of protein utilizing the principle of protein-dye binding. Anal Biochem 72: 248–254. 942051 10.1016/0003-2697(76)90527-3

[27] van Eunen K, Bouwman J, Daran-Lapujade P, Postmus J, Canelas AB, et al (2010) Measuring enzyme activities under standardized in vivo-like conditions for systems biology. Febs J 277: 749–760. 20067525 10.1111/j.1742-4658.2009.07524.x

[28] Bergmeyer HU (1984) Methods of enzymatic analysis (Vol II, III). Deerfield Beach, Florida: Weinheim.

[29] Small JR, Kacser H (1993) Responses of metabolic systems to large changes in enzyme activities and effectors. 1. The linear treatment of unbranched chains. Eur J Biochem 213: 613–624. 8477732 10.1111/j.1432-1033.1993.tb17801.x

[30] Brand IA, Söling HD (1974) Rat liver phosphofructokinase. Purification and characterization of its reaction mechanism. J Biol Chem 249: 7824–7831. 4279251

[31] Grossbard L, Schimke RT (1966) Multiple hexokinases of rat tissues. Purification and comparison of soluble forms. J Biol Chem 241: 3546–3560. 5919684

[32] Kiefer F, Arnold K, Künzli M, Bordoli L, Schwede T (2009) The SWISS-MODEL Repository and associated resources. Nucleic Acids Research 37: D387–D392. 18931379 10.1093/nar/gkn750 PMC2686475

[33] Hubbard SJ, Thornton JM (1993) ‘NACCESS’, Computer Program. Department of Biochemistry and Molecular Biology, University College London.

[34] Raïs B, Puigjaner J, Comín B, Cascante M (1996) Potential errors related to control coefficients determination in mouse muscle glycolysis. In: Westerhoff HV, Snoep JL, Sluse FE, Wijker JE, Kholodenko BN, editors. Biothermokinetics of the Living Cells. Amsterdam: Biothermokinetics Press. pp. 174–180

[35] Marín-Hernández A, Rodríguez-Enríquez S, Vital-González PA, Flores-Rodríguez FL, Macías-Silva M, et al (2006) Determining and understanding the control of glycolysis in fast-growth tumor cells. Flux control by an over-expressed but strongly product-inhibited hexokinase. Febs J 273: 1975–1988. 16640561 10.1111/j.1742-4658.2006.05214.x

[36] Ovadi J, Orosz F (1997) A new concept for control of glycolysis. In: Agius L, Sherratt HSA, editors. Channelling in Intermediary Metabolism London: Portland Press Ltd. pp. 237–268.

[37] Kacser H, Acerenza L (1993) A universal method for achieving increases in metabolite production. Eur J Biochem 216: 361–367. 8375376 10.1111/j.1432-1033.1993.tb18153.x

[38] Fell DA, Thomas S (1995) Physiological control of metabolic flux: the requirement for multisite modulation. Biochem J 311 Pt 1: 35–39. 7575476 10.1042/bj3110035 PMC1136115

[39] Rodriguez-Prados JC, de Atauri P, Maury J, Ortega F, Portais JC, et al (2009) In silico strategy to rationally engineer metabolite production: A case study for threonine in Escherichia coli. Biotechnol Bioeng 103: 609–620. 19219914 10.1002/bit.22271

