# Supplementary material for: Effects of Cadmium and Mercury on the Upper Part of Skeletal Muscle Glycolysis in Mice
Source: PLoS One. 2014 Jan 28;9(1):e80018. doi: 10.1371/journal.pone.0080018 (PMC3904826; doi:10.1371/journal.pone.0080018)
Supplement: Appendix S1 — (DOCX) [file pone.0080018.s001.docx]

**Appendix S1: Derivation of the reaction rate equations for irreversible inhibition of hexokinase and phosphofructokinase by Hg^2+^ and Cd^2+^ added to the cell extract**

Effect of Hg^2+^ on hexokinase and phosphofructokinase kinetic reactions

Here we describe the inhibition of HK and PFK by Hg^2+^ through the mechanism displayed in Fig. 4A: the enzyme can bind mercury at a site distal from the substrate binding site. This binding inactivates the enzyme and is irreversible. Alternatively, mercury binds to the active site. In this case its binding is competitive with the binding of substrate. However, since we pre-incubate the enzyme with the inhibitor in the absence of substrate, the difference is immaterial here.

The inhibitor is pre-incubated with the total cell extract and therefore not only reacts with sulfhydryl groups on HK, but also on an excess of other proteins. We assume that there is an excess (but not a vast excess) of binding sites *E* for Hg^2+^ (inhibitor denoted by *X* and substrate by *S*). The Hg^2+^ therefore disappears in a second order process:


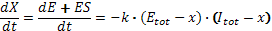
 (A-1)

Here
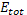
 *E_tot_* represents all protein binding sites for mercury in the extract, *I_tot_*
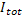
the total amount of inhibitor and *x = EX+ESX = I_tot_ - X*, the amount of enzyme inactivated by the inhibitor. However, we pre-incubated long enough for the extent of inhibition to have become constant, effectively corresponding to an infinite pre-incubation time. This yields:

*x = I_tot_*  (A-2)

The total *active* enzyme concentration (*E_T_*) is represented by the following expression:

$E_{T} = E_{To} - EX-ESX$ (A-3)

Hence the enzyme rate equation obtains the following format:

$v = \frac{k_{2}\cdot E_{To}\cdot\left(1 - \frac{\text{I}_{\text{tot}}}{E_{tot}}\right)\cdot S^{n}}{K_{0.5}^{n} + S^{n}}$ (A-4)

For the purpose of our analysis, equation (A-4) can be reduced to the expression (equation (1)) of the main text:

$v = v^{o}\cdot\left(1 - K_{A}\cdot I_{tot} \right)$ (A-5)

where *v^o^* is the rate expression in the absence of ion metal, i.e.

$v^{o} = k_{2}\cdot E_{To}\cdot S^{n}/\left(K_{0.5}^{n} + S^{n}\right)$ (A-6)

and

$K_{A} = 1/E_{tot}$ (A-7)

Effect of Cd^2+^ on hexokinase and phosphofructokinase kinetic reactions

The inhibition of HK and PFK by the ion metal Cd^2+^ is described by the mechanism displayed in Fig. 4B. *EX_m+1_* and *ES_n_X_m+1_* represent enzyme-metal and substrate-enzyme-metal complexes, with *m* = 1 for HK and *m* = 2 for PFK.

The total active enzyme concentration ($E_{T}$) is given by the following expression:

$E_{T} = E_{To} - EX_{m+1}- ES_{n}X_{m+1}$ (A-8)

As above, for any of the binding sites on a protein, the probability that it has a metal bound is given by *I_tot_/E_tot_*. Hence the probability that *m + 1* binding sites contain the metal is:

$P\left(EX_{m+1}\right) = \left(\frac{I_{tot} }{E_{tot}}\right)^{m+1}$ (A-9)

We assume that the enzyme becomes inactive once all m+1 binding sites have Cd^2+^ bound. As a consequence, this leads to the expression (equation (2)) of the main text:

$v = v^{o}\cdot\left(1 - K_{A}\cdot\left(I_{tot}\right)^{m+1}\right)$ (A-10)

where *v^o^* is the rate expression in the absence of ion metal, i.e.

$v^{o} = k_{2}\cdot E_{To}\cdot S^{n}/\left(K_{0.5}^{n} + S^{n}\right)$ (A-11)
